# Gender differences in factors associated with the health literacy of hospitalized older patients with chronic diseases: A cross-sectional study

**DOI:** 10.3389/fpubh.2022.944103

**Published:** 2022-08-10

**Authors:** Shuting Sun, Jinjin Lu, Yawen Wang, Ya Wang, Lihao Wu, Saiqiong Zhu, Xiuyun Zheng, Xueqin Lu, Hongbo Xu

**Affiliations:** ^1^School of Nursing, Wenzhou Medical University, Wenzhou, China; ^2^Intensive Care Unit, The First Affiliated Hospital of Zhejiang University, Hangzhou, China; ^3^School of Foreign Language Studies, Wenzhou Medical University, Wenzhou, China; ^4^Respiratory Department, The Affiliated Hospital of Wenzhou Medical University, Wenzhou, China; ^5^Endocrinology Department, The Affiliated Hospital of Wenzhou Medical University, Wenzhou, China

**Keywords:** health literacy, gender difference, factors, older patients, chronic diseases

## Abstract

**Background:**

To identify gender differences in factors associated with the health literacy of hospitalized older patients with chronic diseases.

**Methods:**

A total of 471 hospitalized older patients with chronic diseases in four hospitals were investigated from May 2019 to June 2020. The self-developed demographic information questionnaire, the “Health Literacy Scale for Patients with Chronic Diseases” and the “Self-Efficacy for Managing Chronic Diseases 6-item Scale” were applied in this study. Multiple linear regression was used to assess the factors influencing health literacy among older patients with chronic diseases by gender.

**Results:**

The factors influencing health literacy differed by gender. Male health literacy was related to education background, number of children, monthly income, duration of chronic disease and chronic disease self-efficacy. For females, health literacy was associated with age, education background, monthly income, duration of chronic disease and chronic disease treatment.

**Conclusion:**

Healthcare providers should focus on the above-mentioned factors that could help identify those with low health literacy differ base on gender. Gender-specific strategies should be developed to improve the health literacy of older patients with chronic diseases and strengthen their chronic disease management.

## Introduction

Nowadays, the population is growing older. The United Nations reported that 1/6 of the population in the world will be over 65 (16%) by 2050, up from 1/11 in 2019 (9%) (1). In China, there are 264.02 million people aged 60 years or older, accounting for 18.70% (2). Chronic diseases are becoming a predominant public health issue for this population because of their high prevalence (3). In China, the prevalence rate for older adults with chronic diseases was reported to be 75% (4). According to the World Health Organization (WHO) (5), chronic diseases are responsible for almost 71% of deaths worldwide, equivalent to 41 million deaths per year. Therefore, there is an urgent need for healthcare providers to take preventive measures against chronic diseases to alleviate their harm and to enhance the management of chronic diseases in older adults.

Health literacy (HL) is defined as “an individual's ability to obtain, understand, appraise and use basic health information and services to make informed health choices” (6, 7). That is, patients with poor HL tend to be less responsive to health education messages, have difficulty accessing and utilizing health-related information, and are less able to successfully manage chronic diseases (6, 8). Several studies have stated that limited HL may have poor outcomes, including low knowledge, poor chronic disease management, low use of preventive health services and high-risk mortality (9–11). Currently, low HL is a typical occurrence among older patients with chronic diseases (12). In a survey of 264 individuals with heart failure conducted by Cox et al. (13), 33.7% of cases (an average age of 66) had poor HL. Another study found that 76% of 160 older patients with hypertension had inadequate HL (14). Notably, HL is a modifiable determinant of health that can be an effective educational and preventive measure of chronic disease self-management (6, 7, 9, 15). Increasing HL can optimize health outcomes for patients with chronic diseases (16). Thus, to promote healthy aging and strengthen chronic disease management (17, 18), HL of older patients with chronic diseases needs further attention.

As pointed out in the previous studies, there may be physical and sociocultural differences in the health of males and females (19). Individuals tend to differ in their access to, use of health care, and help-seeking behaviors by gender (20). Equally large variability exists among the older population, even more so as they age (19). Hence, HL, which can be identified as a key determinant of health, may vary by gender among the older with chronic diseases (21). Mashi et al. (22) discovered that females with diabetes have lower levels of HL than males. A study toward older patients with heart failure showed that females had a higher prevalence of limited HL than males (15). In addition, Sun (23) assessed the four dimensions of HL (information acquisition ability, communicative interaction ability, health improvement willingness, economic support willingness) in older patients with chronic diseases of both genders. He found that males scored higher in information acquisition ability, and were more adept at understanding and processing information than females. Contrarily, females had better communicative interaction ability than males.

The HL of older patients with chronic diseases is influenced by many factors. Several studies have reported that limited HL was associated with age, low education, family income and chronic disease self-efficacy (24–27). There were also some studies examining the factors that influence HL of males or females with chronic diseases. Research has shown that HL in males was related to age, education, family income and treatment duration (21, 28), while HL in females was associated with age and education (8, 15). However, data on differences in factors influencing HL among older chronic patients from a gender perspective are limited.

Based on the gender perspective, a differentiated strategy will be possible for each gender if the HL's factors can be identified. Thus, this paper attempts to examine gender differences in factors associated with HL, which will identify those with low HL base on gender and provide a basis for developing health education programs to promote the HL of older patients with chronic diseases.

## Materials and methods

### Study design

This research was conducted as a cross-sectional study. The guidelines of Strengthening the Reporting of Observational Studies in Epidemiology (STROBE) were utilized in reporting this study (see Supplementary material).

The ethics committee of the First Affiliated Hospital of Wenzhou Medical University approved the study (approval number: KY2021-104). Informed consent was obtained from all study participants, and they were kept anonymous.

### Sampling methods

A convenience sampling was used for this study. Participants were hospitalized older patients with chronic diseases recruited from four hospitals in Zhejiang Province, China, between May 2019 and June 2020. The inclusion criteria were as follows: (a) having a confirmed diagnosis of one or more chronic diseases (such as chronic obstructive pulmonary disease, hypertension, diabetes, coronary heart disease, peptic ulcer, liver cirrhosis, chronic kidney disease, cerebrovascular disease, etc.); (b) aged 60 years or older; (c) conscious; (d) no neuropsychiatric problem and (e) voluntary participation in this study.

According to Tinsley and Tinsley (29), the sample size should be determined taking into account the number of variables, usually between 1:5 and 10. Therefore, the sample size of this study was estimated based on a sample size of at least 10 times the number of variables. A minimum sample of 122 was calculated using to the following parameters: first, the number of variables in this study was 11; second, an attrition rate of the questionnaires was assumed at 10% and last, the calculation formula was (11^*^10) / (1–10%). Besides, we used GPower 3.1.9.7 (30), an automated sample size calculating software. *T*-tests and the mean difference between two independent means (two groups) were selected. To ensure adequate test power (1-β = 0.95, α = 0.05) and moderate effect size (*d* = 0.5), a sample size of 210 was suggested for this study. Then, considering a 10%, the sample size was inflated to 233 cases. In conclusion, the larger one was selected and at least 233 participants were included.

### Study measures

#### Demographic characteristics

According to the research purpose, demographic variables including gender, age, marital status, education, number of children, living type, monthly income, number of chronic diseases, duration of chronic diseases, and chronic disease treatment, were collected. Monthly income (Chinese Yuan) was reported as the per capita monthly income of households. The duration of chronic diseases was divided into <10 years and ≥10 years. This grading standard is mainly based on the high incidence time of complications from common chronic diseases (31, 32).

#### Health literacy scale for patients with chronic diseases

The “Health Literacy Scale for Patients with Chronic Diseases,” developed by Jordan et al. (33) and translated by Sun (23), was applied to assess HL. The instrument is a 24-item self-report questionnaire with four domains: information acquisition ability, communicative interaction ability, health improvement willingness and economic support willingness. Each item is scored on a 5-point scale (1 = absolutely impossible to 5 = no problem), with a total score of 120. The higher the total score, the higher the patient's HL. It is the first HL scale for chronic disease patients in China, which comprehensively reflects the content of HL. The Cronbach's α for each dimension of the scale ranged from 0.885 to 0.925, and the test-retest reliability was 0.683. In this study, the Cronbach's α of the scale was 0.815, and the test-retest reliability was 0.734.

#### Self-efficacy for managing chronic diseases 6-item scale

This research drew on the Self-Efficacy for Managing Chronic Diseases 6-item Scale developed by Lorig et al. (34), which consists of six items. Scores for each item range from 1 (no confidence) to 10 (the highest self-confidence). The average score of the six items represents the patient's self-efficacy level, with higher scores indicating greater self-efficacy.

According to the index rating, self-efficacy is classified into three levels: low (<5 points), medium (5–7 points), and high (≥7 points) (35). The Cronbach's α of each dimension was 0.77–0.92, and the test-retest reliability was 0.72–0.89. In this study, the Cronbach's α was 0.938, and the test-retest reliability was 0.855.

### Data collection

Before the investigation, informed consent was obtained from the nursing department of the four hospitals, and the interviewers had received uniform training to ensure homogeneity of survey skills. During the formal survey, the interviewers first explained the purpose, significance, content, and anonymity of the research to all eligible patients to obtain their consent. Next, information was collected through face-to-face interviews. For those patients who had difficulty or were unable to complete the questionnaire by themselves, the interviewers would give neutral word explanations or write on their behalf to ensure that the records were consistent with the patients' options.

**Figure 1:**
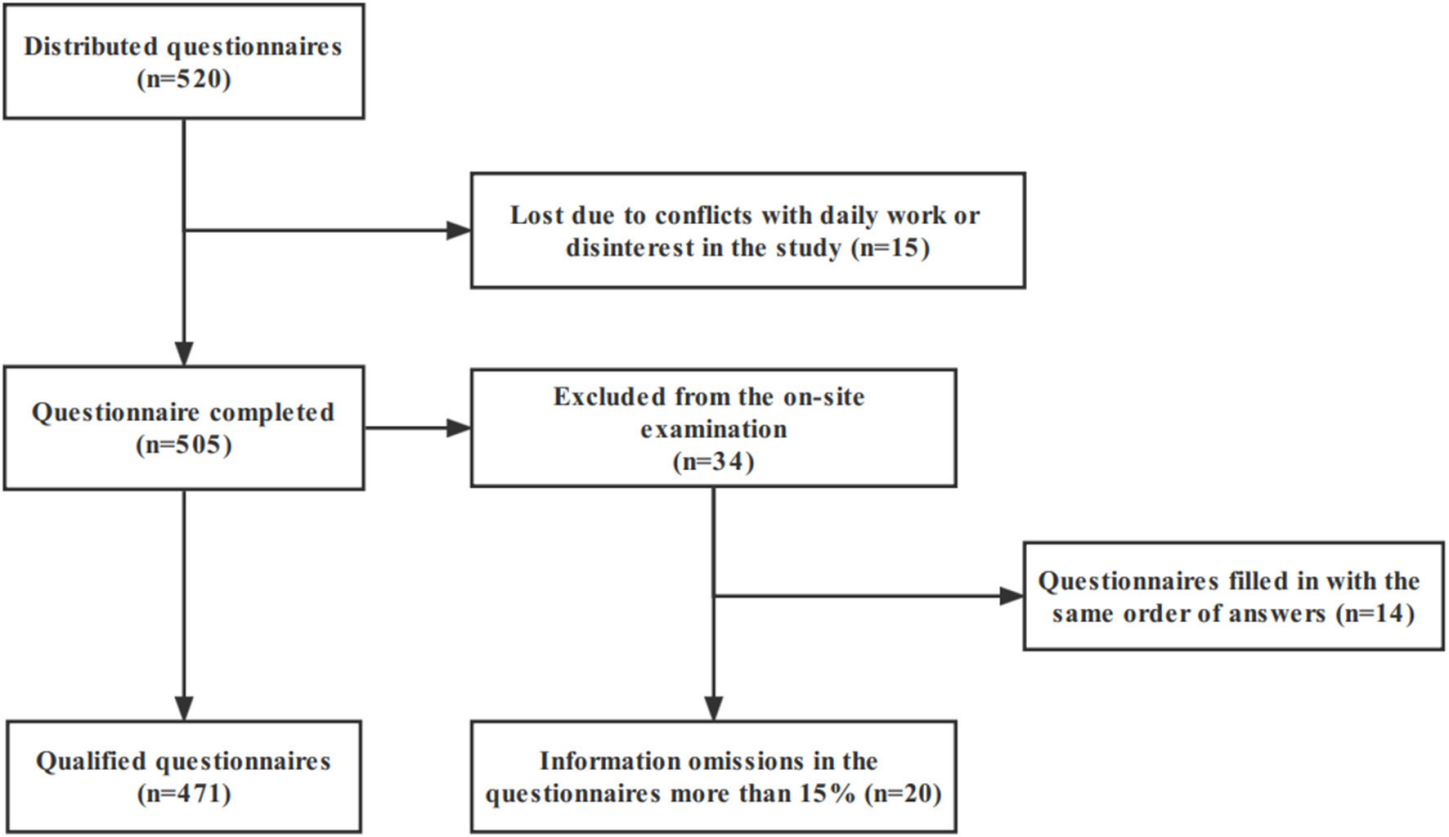
Flowchart of the study population.

In this study, a total of 520 questionnaires were distributed, and 15 cases were lost due to conflicts with daily work or disinterest in the study, with an attrition rate of 2.88%. The remaining 505 patients completed the questionnaire, and a total of 34 cases were excluded from the on-site examination because the information omissions in the questionnaires more than 15% (*N* = 20) or in the same order (*N* = 14), which the researchers believe may have been perfunctory on the part of the respondents. Finally, 471 completed questionnaires were obtained, with an efficiency rate of 90.58% (Figure 1).

### Statistical analyses

All statistical analyses were performed using SPSS 25.0. Numerical variables were computed as mean and standard deviation, and categorical variables were calculated as number and percentages (%). Gender differences in patient characteristics and HL were investigated by using independent samples *t*-test (numerical variables), one-way analysis of variance (numerical variables), and chi-squared tests (categorical variables). Subsequently, we used multiple linear regression models to assess gender differences in factors associated with HL among older patients with chronic diseases. The inspection level was set at α = 0.05. Missing quantitative information was replaced by the mean, and missing categorical data were replaced by the mode (36).

## Results

### Demographic characteristics of older patients

The demographic characteristics are listed in Table 1. Of the 471 hospitalized older patients with chronic diseases, 240 (51%) were females. The average age was 73.22 (SD = 8.534). Most patients (84.7%) were married, while 15.3% were divorced or widowed. Concerning educational level, 75 (15.9%) received a high school education or above. In terms of living type, most patients (84.7%) lived with family members, and 65.6% had more than three children. Furthermore, 83.5% of the sample had a monthly income of <5,000 Chinese Yuan. A total of 273 (58%) had two or more chronic diseases, and 219 (46.5%) had chronic diseases for more than 10 years. Regarding chronic disease treatment, 336 (71.3%) chose all-Western medicine.

**Table 1:** Descriptive statistical analysis in demographic characteristics.

**Variables**	**Total** ** (*n* = 471)**	**Male** ** (*n* = 231)**	**Female** ** (*n* = 240)**	***P***
	***n* (%)**	***n* (%)**	***n* (%)**	
**Age (year)**				
60–69	183 (38.9)	78 (33.8)	105 (43.8)	**0.043**
70–79	195 (41.4)	99 (42.9)	96 (40.0)	
≥80	93 (19.7)	54 (23.4)	39 (16.3)	
**Marriage**				
Married	399 (84.7)	207 (89.6)	192 (80.0)	**0.004**
Divorced or widowed	72 (15.3)	24 (10.4)	48 (20.0)	
**Education**				
Illiterate	165 (35.0)	66 (28.6)	99 (41.3)	**0.001**
Elementary school	189 (40.1)	102 (44.2)	87 (36.3)	
Middle school	42 (8.9)	15 (6.5)	27 (11.3)	
High school or above	75 (15.9)	48 (20.8)	27 (11.3)	
**Number of children**				
≤ 2 children	162 (34.4)	78 (33.8)	84 (35.0)	0.778
>3 children	309 (65.6)	153 (66.2)	156 (65.0)	
**Living type**				
Live with family members	399 (84.7)	204 (88.3)	195 (81.3)	**0.033**
Live alone	72 (15.3)	27 (11.7)	45 (18.8)	
Monthly income (CNY)^*^				
<1,500	54 (11.5)	21 (9.1)	33 (13.8)	**0.001**
1,501–3,000	168 (35.7)	78 (33.8)	90 (37.5)	
3,001–5,000	171 (36.3)	78 (33.8)	93 (38.8)	
>5,000	78 (16.6)	54 (23.4)	24 (10.0)	
**Number of chronic diseases**				
1	198 (42.0)	99 (42.9)	99 (41.3)	0.724
≥2	273 (58.0)	132 (57.1)	141 (58.8)	
**Duration of chronic disease (years)**				
<10	252 (53.5)	108 (46.8)	144 (60.0)	**0.004**
≥10	219 (46.5)	123 (53.2)	96 (40.0)	
**Chronic disease treatment**				
All-Western medicine	336 (71.3)	159 (68.8)	177 (73.8)	0.238
Chinese and western medicine	135 (28.7)	72 (31.2)	63 (26.3)	

* *1000 CNY= 147.40 EUR / 149.12 USD*.

*The bold values indicated P < 0.05*.

### Gender differences in health literacy

As shown in Table 2, there was no difference in the total scores of HL and the four dimensions between male and female older patients (*P* > 0.05).

**Table 2:** Gender differences in health literacy (M ± SD).

**Variables**	**Male (*n* = 231)**	**Female (*n* = 240)**	***P***
Total score of health literacy	82.05 ± 24.72	81.45 ± 20.73	0.774
Information acquisition ability	27.51 ± 10.63	26.71 ± 9.04	0.377
Communicative interaction ability	31.04 ± 10.03	31.30 ± 8.40	0.759
Health improvement willingness	16.31 ± 4.16	16.04 ± 4.10	0.471
Economic support willingness	7.01 ± 2.71	7.19 ± 2.50	0.467

### Differences in health literacy among older patients with different characteristics

Through univariate analysis, significant differences in HL were found among patients in terms of education, number of children, monthly income, chronic disease treatment, and chronic disease self-efficacy (*P* < 0.05). Specifically, patients with higher educational level, ≤ 2 children, higher monthly income, all-Western medicine and patients with higher chronic disease self-efficacy had higher HL score. Additionally, there were marginal statistical significance in the aspects of age and the duration of chronic disease (*P* < 0.10). The result showed patients who were older and had chronic conditions ≥10 years had lower HL scores (see Table 3).

**Table 3:** Difference in health literacy among older patients with different characteristics.

**Variables**	**M ±SD**	***F*/*t***	***P***
**Age (year)**			
60–69	84.66 ± 18.78	2.918	0.055
70–79	80.77 ± 23.06		
≥80	78.06 ± 28.14		
**Marriage**			
Married	82.41 ± 22.57	1.486	0.138
Divorced or widowed	78.08 ± 23.56		
**Education**			
Illiterate	69.73 ± 23.36	45.269	**<0.001**
Elementary school	82.75 ± 16.61		
Middle school	90.00 ± 25.79		
High school or above	101.04 ± 16.39		
**Number of children**			
≤ 2 children	91.28 ± 19.66	6.903	**<0.001**
>3 children	76.75 ± 22.69		
**Living type**			
Live with family members	81.48 ± 23.18	−0.592	0.554
Live alone	83.21 ± 20.31		
**Monthly income (CNY)**			
<1,500	68.83 ± 13.10	55.459	**<0.001**
1,501–3,000	70.34 ± 20.22		
3,001–5,000	89.04 ± 20.95		
>5,000	99.27 ± 18.86		
**Number of chronic diseases**			
1	82.41 ± 21.61	0.539	0.590
≥2	81.26 ± 23.57		
**Chronic disease duration (years)**			
<10	83.57 ± 20.84	1.852	0.065
≥10	79.64 ± 24.65		
**Chronic disease treatment**			
All-Western medicine	85.38 ± 20.10	5.054	**<0.001**
Chinese and western medicine	72.69 ± 26.26		
**Chronic disease self-efficacy**			
Low	58.25 ± 27.29	31.679	**<0.001**
Medium	77.79 ± 20.37		
High	86.61 ± 21.08		

*The bold values indicated P <0.05*.

### Gender differences in influencing factors of patients' health literacy

Taking the HL scores of males and females as the dependent variables, all independent variables were incorporated into the model, regardless of their statistical significance in univariate analysis (37). The enter method was used for regression analysis (α_in_ = 0.05, α_out_ = 0.10). After adjusting for covariates, the results showed that HL was closely related to education background, number of children, monthly income, duration of chronic disease and chronic disease self-efficacy for males. The details were as follows: First, educational background has a positive impact on HL, among which the HL levels of older males with elementary school, middle school, high school and above degrees were significantly higher than those of illiterate males (B = 14.122, SE = 3.358, *p*-value = 0.000; B = 22.760, SE = 5.648, *p*-value = 0.000; B = 18.939, SE = 4.559, *p*-value = 0.000). Second, the HL scores of the males with more than 3 children were significantly lower than those with ≤ 2 children (B = −7.277, SE = 2.859, *p*-value = 0.012). Third, monthly income had a positive effect on HL, that is, the higher the income was, the higher the HL level. In particular, those with incomes of 3,001–5,000 and >5,000 had significantly higher HL scores than those with incomes below 1,500 (B = 18.591, SE = 4.605, *p*-value = 0.000; B = 26.293, SE = 5.195, *p*-value = 0.000). Fourth, those with chronic disease duration ≥10 years had significantly higher HL scores compared with those with a duration <10 years (B = 6.132, SE = 2.646, *p*-value = 0.021). Fifth, chronic disease self-efficacy has a positive impact on HL, especially the HL score of males with high self-efficacy, which is significantly higher than that of males with low self-efficacy (B = 17.652, SE = 4.539, *p*-value = 0.000). The model explained 50.6% (*R*^2^ = 0.506, adjusted *R*^2^ = 0.476) of the variance in the total HL score of males.

Among older females, HL was strongly associated with age, education background, monthly income, duration of chronic disease and chronic disease treatment. The details were as follows: First, compared to patients aged 60–69 years, females aged ≥80 years had significantly lower literacy scores (B = −22.949, SE = 3.206, *p*-value = 0.000). Second, educational background has a positive impact on HL, among which the HL levels of older females with high school and above degrees were significantly higher than those of illiterate (B = 24.278, SE = 3.772, *p*-value = 0.000). Third, monthly income had a positive effect on HL; that is, the higher the income was, the higher the HL level. Those with incomes of 1,501–3,000, 3,001–5,000 and >5,000 had significantly higher HL scores than those with incomes below 1,500 (B =7.538, SE = 3.101, *p*-value = 0.015; B = 20.203, SE = 3.312, *p*-value = 0.000; B = 12.916, SE = 4.494, *p*-value = 0.004). Fourth, those with chronic disease duration ≥10 years had significantly lower literacy scores compared to those with chronic disease duration <10 years (B = −4.829, SE = 2.041, *p*-value = 0.019). Fifth, the HL scores of the females treated with all-Western medicine were significantly higher than those treated with a combination of Western and Chinese medicine (B = −9.459, SE = 2.568, *p*-value = 0.000). The model explained 53.7% (*R*^2^ = 0.537, adjusted *R*^2^ = 0.510) of the variance in the total score of female HL (see Table 4).

**Table 4:** Gender differences in influencing factors of patients' health literacy using multiple linear regression.

	**Male** ^**†**^			**Female** ^**‡**^		
	**B**	**SE**	***P***	**B**	**SE**	***P***
(Constant)	47.036	10.228	0.000	88.779	8.742	0.000
**Age (year)**						
60–69	Ref	Ref	Ref	Ref	Ref	Ref
70–79	3.981	2.909	0.173	−2.959	2.156	0.171
≥80	5.723	3.793	0.133	−22.949	3.206	**0.000**
**Education**						
Illiterate	Ref	Ref	Ref	Ref	Ref	Ref
Elementary school	14.122	3.358	**0.000**	3.144	2.265	0.166
Middle school	22.760	5.648	**0.000**	6.534	3.778	0.085
High school or above	18.939	4.559	**0.000**	24.278	3.772	**0.000**
**Number of children**						
>3 (Ref: ≤ 2 )	−7.277	2.859	**0.012**	−3.781	2.646	0.154
**Monthly income (CNY)**						
<1,500	Ref	Ref	Ref	Ref	Ref	Ref
1,501–3,000	7.375	4.639	0.113	7.583	3.101	**0.015**
3,001–5,000	18.591	4.605	**0.000**	20.203	3.312	**0.000**
>5,000	26.293	5.195	**0.000**	12.916	4.494	**0.004**
**Chronic disease duration (years)**						
≥10 (Ref: <10)	6.131	2.646	**0.021**	−4.829	2.041	**0.019**
**Chronic disease treatment**						
All-Western medicine (Ref: Chinese and western medicine)	−4.400	2.946	0.137	−9.459	2.568	**0.000**
**Chronic disease self-efficacy**						
Low	Ref	Ref	Ref	Ref	Ref	Ref
Medium	8.876	5.083	0.082	7.055	4.765	0.140
High	17.652	4.539	**0.000**	5.908	4.834	0.223

† *R^2^ = 0.506, Adjusted R^2^ = 0.476*.

‡ *R^2^ = 0.537, Adjusted R^2^ = 0.510*.

*The bold values indicated P < 0.05*.

## Discussion

The study illuminated the HL and affecting factors among hospitalized older patients with chronic diseases of different genders. Our results suggested that education background, number of children, monthly income, duration of chronic disease and chronic disease self-efficacy were significant factors associated with HL in men. The factors affecting HL in women included age, education background, monthly income, duration of chronic disease and chronic disease treatment. The above findings could lay the groundwork gender-based HL interventions in the future.

In this study, the HL of older patients with chronic diseases generally at a lower level, which indicates the HL needs to be improved. Although the HL scores of males were slightly higher than those of females, the difference was not statistically significant, in line with the results of Peterson et al. (24) and Lin and Xiao (38). However, the finding was inconsistent with the results of Lee and Son (8), which suggested that older female patients have lower HL than males. The possible explanation for the difference lies in the demographic characteristics and disease-related information of the two research samples.

Notably, this study revealed that there are similarities and differences in the influencing factors of HL by gender. Education background, monthly income, and chronic disease duration were associated with HL in both males and females. Education background had a significant positive predictive effect on HL, independent of gender. Several studies have arrived at similar conclusions (8, 25, 39). The possible reason is that education is the foundation for HL (40) and educated older patients have strong learning and comprehension skills (26). They will actively absorb and use relevant information in interaction with doctors in order to better promote the rehabilitation of chronic diseases (38). Therefore, the results may suggest there is a need for easy-to-understand health education for low-educated patients to improve their HL. In terms of monthly income, this finding was in agreement with Lin and Xiao (38) and Schaeffer et al. (39), who found that it was correlated with HL. Older patients with higher income expressed a strong willingness to health improvement after meeting their daily basic needs (23). They tend to invest more money into their own health management and access primary care earlier, which might explain their better HL (23). Instead, to guarantee material lives, patients with less income barely have time and extra money to take care of their own health, which limits patients' contact with health information (41). Additionally, this study discovered that the duration of chronic diseases was one of the influencing factors, similar to prior studies (39, 42). Different from the above two papers, which showed that the duration of chronic diseases was positively correlated with HL, we found that chronic disease duration has a positive effect on HL in males but a negative effect on HL in females. The reason for this might be that males usually have higher information acquisition ability, and are better at understanding and processing information than females (23). As the duration of disease prolonged, men may accumulate more knowledge about disease management and be more experienced in coping with disease. For older women, their ability to access, appraise and utilize health information becomes increasingly inadequate as the disease progresses and may worsen, thereby weakening their HL levels.

The study also confirmed that the number of children and chronic disease self-efficacy had a significant impact on older males' HL (*P* < 0.05). However, the above two factors had no influence on HL for females. Specifically, males with > 3 children had lower HL than those with ≤ 2 children. This finding differs from Yuan et al. 's (26) research but is somewhat similar to that of Hu et al. (43), who found that a family size of more than 4 was a risk factor for HL among older adults. The increase in the number of children results in high economic pressure and resource strain on families. Considering traditional Chinese society, men still follow the pattern that men play a key role in society while women are confined to family chores (44). Males are the main workforce of a family during their youth and maturity, while women mostly rely on family income (44, 45). Accordingly, males with more children may encounter higher professional pressure than females, which limits their access to critical health-promoting resources in the long run. Furthermore, chronic disease self-efficacy was more likely to positively affect males' HL. This result was in discordance with previous studies, which have shown that HL was positively correlated with chronic disease self-efficacy independent of gender (25, 46). It has been reported that education can positively predict self-efficacy (47). This may be related to the mediating effect of education on the path of self-efficacy to HL. In this study, a higher proportion of males than females had high school and above education, so males with high chronic disease self-efficacy may have better HL. However, there remains insufficient argument to explain this result, which needs to be further verified. Given the factors involved, more attention should be paid to older chronic disease males with many children and low self-efficacy.

For females, age and chronic disease treatment were the main factors for HL. This result found that low HL occurred in older females, which was similar to previous studies (48, 49). In view of unique historical reasons and the Chinese cultural context, there has been a considerable gender difference in educational attainment between males and females, and older females' educational attainment is generally limited (50). Higher educational attainment is linked to adequate HL (51). Therefore, this can undermine the ability of older women to understand and adhere health information, such as barriers to communication with physicians (52). In contrast, younger female patients tend to have a higher education level, as well as superior communication and comprehension skills. As a result, they respond effectively to health education messages and fully comply with physician recommendations. However, the impact of different eras on males' receipt of education was not immediately obvious. Thus, our findings indicate that age has no influence on males' HL. It is important to assess the age of older females with chronic diseases while offering health education. Simultaneously, educational equity may be an important factor in promoting the HL of females with chronic diseases and narrowing the gender gap. Besides, the study suggested that chronic disease treatment was an independent risk factor for HL in female patients. Females treated with all-Western medicine had a greater degree of HL than those treated with a mix of traditional Chinese and Western medicine. Local patients who preferred herbal medicine as an adjuvant therapy tended to be older and less educated. Consequently, their overall HL levels, such as the ability to obtain health resources, may have been skewed.

## Limitations

There are several limitations in this paper. First, convenience sampling was conducted to select participants in this study, which may have a certain impact on the representativeness of the sample. Second, the HL and self-efficacy were based on participants' self-reported data, which might have self-reported bias. Finally, because it is a cross-sectional study, HL and influencing factors among older patients with chronic diseases of different genders should be demonstrated as association rather than causation.

## Conclusion

An aging society and the growing prevalence of chronic diseases have led to increasing health concerns. The study highlights the importance of gender in improving HL among older patients with chronic diseases. The HL of male hospitalized patients with chronic diseases was linked to education background, number of children, monthly income, duration of chronic disease and chronic disease self-efficacy. Female HL was correlated with age, education background, monthly income, duration of chronic disease and chronic disease treatment. This paper can help healthcare providers realize that gender differences cannot be ignored in improving the HL of older patients with chronic diseases. When assessing HL of older patients with chronic diseases, healthcare providers should focus on the above-mentioned factors that could help identify those with low HL differ base on gender. Gender-specific strategies should be developed and appropriate measures should be taken to maximize their HL and promote healthy aging. Moreover, all education delivery should be delivered in a manner appropriate to individual HL (16).

## Data availability statement

The original contributions presented in the study are included in the article/Supplementary material, further inquiries can be directed to the corresponding authors.

## Ethics statement

Written informed consent was obtained from the individual(s) for the publication of any potentially identifiable images or data included in this article.

## Author contributions

HX, XL, and SS: study design. SS, JL, YaW, XL, and XZ: data collection. JL, YawW, and SZ: data analysis. HX, XL, and XZ: study supervision. SS, JL, LW, and HX: manuscript writing. All authors contributed to the article and approved the submitted version.

## Funding

This research was supported by Humanities and Social Science Project of Chinese Ministry of Education (15YJCZH196).

## Conflict of interest

The authors declare that the research was conducted in the absence of any commercial or financial relationships that could be construed as a potential conflict of interest.

## Publisher's note

All claims expressed in this article are solely those of the authors and do not necessarily represent those of their affiliated organizations, or those of the publisher, the editors and the reviewers. Any product that may be evaluated in this article, or claim that may be made by its manufacturer, is not guaranteed or endorsed by the publisher.
